# Managing Brugada Syndrome in a Private Dental Practice: A Structured Case-Based Review

**DOI:** 10.7759/cureus.88977

**Published:** 2025-07-29

**Authors:** Francesco Valente, Giacomo Gerboni, Pierluigi Valente, Lapo Sbrenna, Andrea Sbrenna

**Affiliations:** 1 Oral Implantology, San Damiano Dental Clinic, Rome, ITA; 2 Oral Medicine, San Damiano Dental Clinic, Rome, ITA; 3 Dentistry, School of Dentistry, Vita-Salute San Raffaele University, Milano, ITA; 4 Dentistry, School of Dentistry, University of Bologna, Bologna, ITA; 5 Private Practice, Humanis Dental Center, Perugia, ITA

**Keywords:** adrenaline, articaine, brugada, brugada dental care, brugada syndrome, dental anesthesia, epinephrine, lidocaine, regional anesthetics

## Abstract

Brugada syndrome (BrS) is a rare inherited cardiac condition associated with a heightened risk of malignant arrhythmias, particularly during exposure to various pharmacological agents, including certain local anesthetics with sodium channel-blocking properties. This condition often generates significant concern among dental professionals, as the routine use of local anesthetics raises uncertainty about safety protocols and perceived medico-legal risks, frequently leading to patient refusal. The result is a silent yet systematic exclusion of these patients from standard pathways of care, with implications that extend beyond the clinical domain to encompass ethical, deontological, and social dimensions. We report the case of a 29-year-old male with BrS and a subcutaneous implantable cardioverter-defibrillator who underwent a conservative restorative dental procedure in a private outpatient clinic. Given the potential risk of arrhythmia during anesthetic infiltration, 4% articaine with epinephrine 1:100,000 was administered slowly and at the minimum effective dose under continuous cardiologic supervision, with real-time ECG and vital sign monitoring initiated prior to injection and maintained throughout the session. The treatment was completed uneventfully, with no ECG abnormalities observed. While limited to a single observation, this case illustrates that, under carefully controlled conditions and with appropriate precautions, outpatient dental management of patients with BrS may be considered a viable option in selected cases. This report includes a brief review of the literature and a focused discussion on the medico-legal implications to contextualize this case and support safe, ethical, and reproducible clinical decision-making in similar outpatient settings.

## Introduction

Brugada syndrome (BrS) is an inherited cardiac arrhythmia disorder associated with an increased risk of sudden cardiac death due to ventricular fibrillation, particularly in structurally normal hearts. The global prevalence of BrS varies widely, with a pooled estimate of approximately 0.5 cases per 1,000 individuals, and the highest prevalence is reported in Southeast Asia, reaching up to 1.8 cases per 1,000 population [1]. Although BrS remains clinically silent in many individuals, its public health relevance is underscored by its association with sudden cardiac death, particularly in younger populations. It is characterized by a distinctive electrocardiographic (ECG) pattern showing coved-type ST-segment elevation in the right precordial leads (V1-V3), often in association with sodium channel dysfunction. Although commonly attributed to the work of the Brugada brothers [2], who formally defined the syndrome in 1992, the first documented descriptions of this clinical entity were reported four years earlier by Italian authors Andrea Nava and his colleagues Bortolo Martini, Bruno Canciani, Gianfranco Buja, Luciano Daliento, and Gaetano Thiene. In 1988, they published a series of studies in both national and international journals, including Giornale Italiano di Cardiologia, Mises à Jour Cardiologiques, and the American Heart Journal, describing cases of ventricular fibrillation in patients without structural heart disease, associated with characteristic ECG patterns. BrS is most frequently linked to mutations in the SCN5A gene, affecting cardiac sodium channel function [3]. Genetic testing for SCN5A mutations, once a pathogenic variant is identified in the proband, enables cascade screening of family members as part of a family-centered approach to early identification and tailored clinical follow-up. Clinical manifestations may be silent or triggered by fever, vagal tone, or certain medications, including local anesthetics with sodium channel-blocking properties. This raises particular concern in dental settings, where such agents are routinely used. BrS is a potentially life-threatening cardiac channelopathy associated with an increased risk of sudden arrhythmic death, and, due to its electrophysiological features and the common indication for implantable cardioverter-defibrillators, it is generally classified as ASA Physical Status III. ASA III refers to the American Society of Anesthesiologists Physical Status Classification System and denotes a patient with severe systemic disease that is not life-threatening but may limit activity and require caution during medical or surgical procedures. While ASA classification has traditionally been the responsibility of anesthesiologists, current evidence suggests that surgeons often assign ASA classes during the preoperative planning phase [4]. In light of this, complex or high-risk cases, such as those involving BrS, may benefit from a multidisciplinary preoperative discussion. Involving the physician most familiar with the patient’s cardiac history, typically the treating cardiologist, may help ensure a more accurate and collaborative risk assessment, particularly in settings where anesthesiologist input is not available. However, this assessment may vary depending on the patient’s actual clinical status and degree of disease control. From a medico-legal standpoint, the assignment of an ASA class has practical implications for procedural settings and monitoring requirements, especially in non-hospital facilities. While ASA classification is formally the responsibility of the anesthesiologist when anesthesia is administered, in the absence of active anesthetic involvement, risk assessment may be delegated to the specialist managing the underlying disease, such as the cardiologist, who can certify clinical stability and support outpatient treatment decisions [5]. In real-world practice, nearly all Italian patients with BrS receive dental care in private outpatient settings rather than in hospital-based environments. This trend also reflects broader systemic and sociocultural dynamics. According to a recent report by the Italian National Association of Dentists (ANDI), approximately 64,000 dentists are currently licensed in Italy. Among them, an estimated 3,457 provide services within the National Health Service (Servizio Sanitario Nazionale, SSN), primarily under contractual agreements, while only 1,094 are employed on a full-time basis. Consequently, approximately 93% of Italian dentists practice in the private sector, predominantly as self-employed professionals, with only around 7% operating within the public healthcare system. As a result, hospital-based dental services are rarely accessible or entirely unavailable to most patients, including those with complex cardiovascular conditions. When available, such services within hospital settings are typically limited to emergency dental care. This structural imbalance between the public and private sectors implies that the vast majority of dental services are, in fact, performed in private facilities, which are generally more accessible, more flexible in scheduling, and frequently equipped with advanced clinical technologies. Moreover, patients retain the legal right to choose their healthcare provider, a principle particularly relevant in dentistry, where care is often longitudinal in nature and may have been initiated well before the diagnosis of BrS. Therefore, when individualized assessment indicates a manageable clinical profile and institutional policies do not preclude outpatient treatment, private dental clinics may represent a reasonable and practicable setting for dental care in patients with inherited channelopathies. This observation has been corroborated through the analysis of online patient communities, discussion forums, and social media groups dedicated to BrS including the Italian forum https://sindromedibrugada.forumfree.it, private groups such as “Sindrome di Brugada” (Italian-speaking, with over 4,200 members), and the international English-speaking groups “Brugada Syndrome Support Group” (over 2,600 members), “Brugada Syndrome Awareness” (over 2,300 members), and “Brugada” (over 3,200 members). All online resources were accessed and verified in June 2025. This real-world scenario underscores the need for greater clinical and scientific clarification on the safe dental management of BrS patients and provides the rationale for the present manuscript. Regional anesthesia is integral to dental care, but in BrS patients, it requires careful consideration. Among local anesthetics, lidocaine and articaine, both amide-type agents, are currently the only substances for which the literature provides some evidence suggesting relative safety in BrS patients, particularly when administered in low doses with epinephrine to limit systemic absorption [6]. The absence of robust clinical trials specifically addressing local anesthetic safety in BrS continues to pose a challenge for dental clinicians, who must rely on case-based evidence and expert recommendations. As a result, treatment decisions must rely on individualized, multidisciplinary risk assessment, involving close collaboration between dentists and cardiologists, and should be carried out under controlled clinical conditions to minimize potential arrhythmogenic triggers. Reference tools such as BrugadaDrugs.org and case series such as those published by Dell’Olio et al. in 2021 have begun to offer structured recommendations, though more clinical data are needed [7]. This report presents the case of a 29-year-old male with BrS and a subcutaneous implantable cardioverter-defibrillator (S-ICD), who safely underwent a conservative restorative dental procedure under local anesthesia, performed in a private clinical setting, with real-time supervision by a cardiology specialist and continuous ECG monitoring. A literature review is also included to contextualize the management strategy and support the development of standardized perioperative protocols for BrS patients in dentistry. This report also addresses the potential medico-legal implications associated with the management of BrS patients in outpatient settings, emphasizing the importance of aligning individualized clinical risk stratification with current legal and regulatory frameworks.

This case report is based on routine clinical documentation and observational findings and does not constitute research involving human subjects under current institutional and regulatory definitions. In accordance with institutional policy, the case was deemed exempt from formal IRB/Ethics Committee review. Written informed consent was obtained from the patient for the dental treatment and for publication of this report and any accompanying images. The ECG images included do not contain any data that could lead to patient identification.

## Case presentation

**Figure 1:**
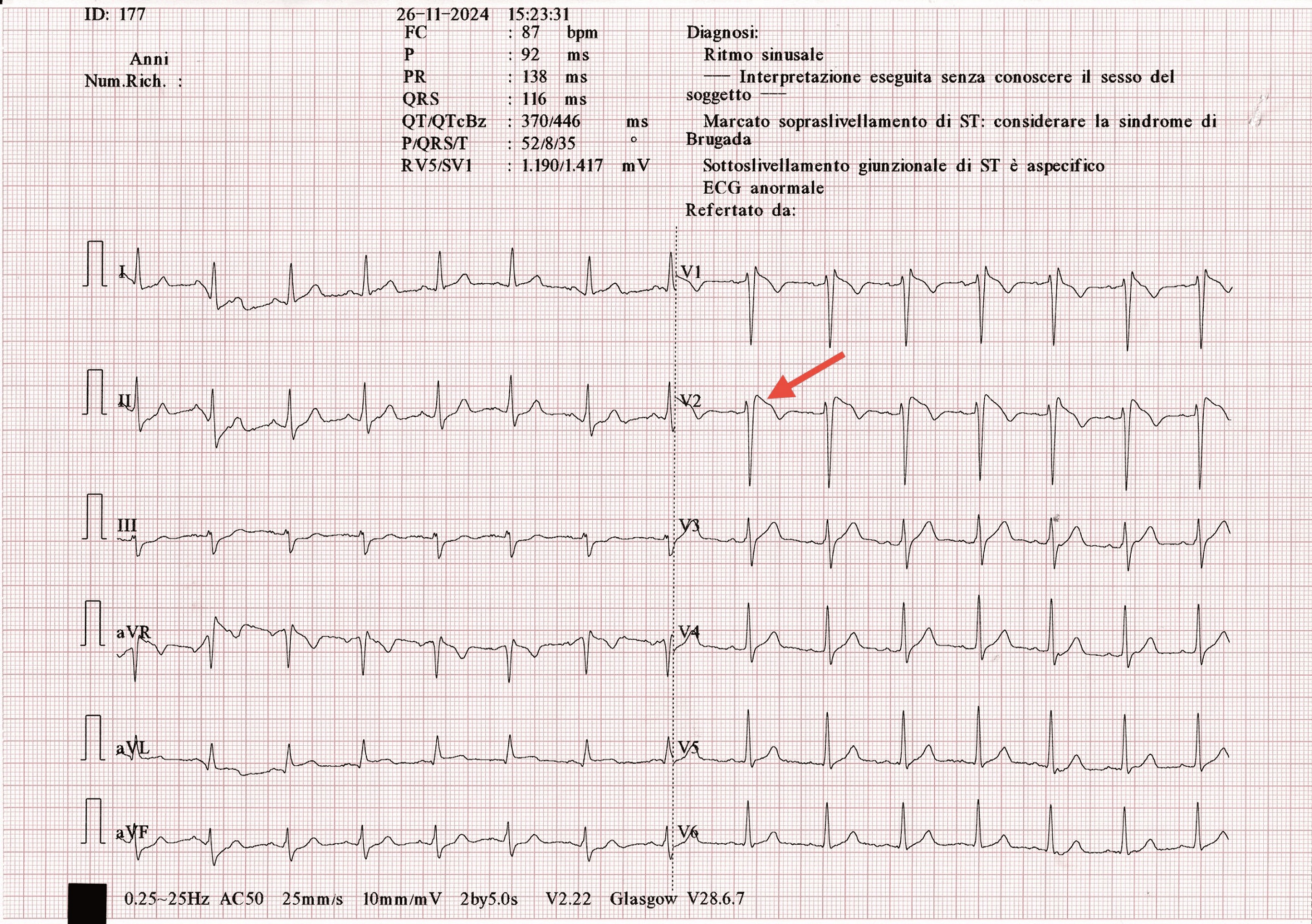
A 12-lead ECG showing a coved-type ST-segment elevation ≥2 mm in lead V2 (red arrow), followed by a negative T wave, consistent with a spontaneous Brugada type 1 pattern. The tracing was recorded at rest and revealed sinus rhythm at 87 bpm, normal atrioventricular conduction (PR 138 ms), a mildly prolonged QRS duration (116 ms), and QTc of 446 ms.

A 29-year-old male patient presented for a dental examination at our clinic, San Damiano Dental Center in Rome, Italy, in June 2025, complaining of pain involving the upper left first molar. The treatment was considered quite urgent, as the patient had been in this state of dental deterioration for many years, resulting in a deep carious lesion approaching the dental pulp. During the anamnesis, it emerged that the patient had received an initial diagnosis of BrS in 2019, at the age of 23, after experiencing a syncopal episode on his way to work, which had led to emergency hospitalization and urgent surgery for pneumothorax. No cases of sudden death have been reported among his family members. A diagnostic flecainide provocation test subsequently revealed a type 1 Brugada ECG pattern, thus confirming the diagnosis. He subsequently underwent implantation of an S-ICD (EMBLEM™ MRI S-ICD System, Boston Scientific S.p.A., Milan, Italy). To date, one case of shock has been documented by the implanted S-ICD; however, no treated or untreated arrhythmic episodes have been recorded, suggesting that the shock likely corresponds to the defibrillation test routinely performed at the time of implantation. These data were obtained from the official device interrogation report provided directly by the patient. The patient sought treatment at our specialized facility in Rome after facing difficulty in his hometown finding a dentist willing to proceed, likely due to concerns about anesthetic risks in BrS. Due to extensive carious damage, the tooth required caries removal, root canal treatment, core build-up, and intraoral scanning for CAD/CAM crown fabrication. In light of the patient’s underlying arrhythmogenic disorder and the anticipated need for local anesthetic administration during the restorative dental procedure, a comprehensive preoperative cardiologic assessment was undertaken. This included the review of a standard 12-lead electrocardiogram, performed eight months earlier by the patient’s cardiologist, and direct liaison with the same specialist to evaluate recent clinical findings, assess arrhythmic risk stratification, and confirm both the stability and functional status of the implanted S-ICD (Figure 1).

**Figure 2:**
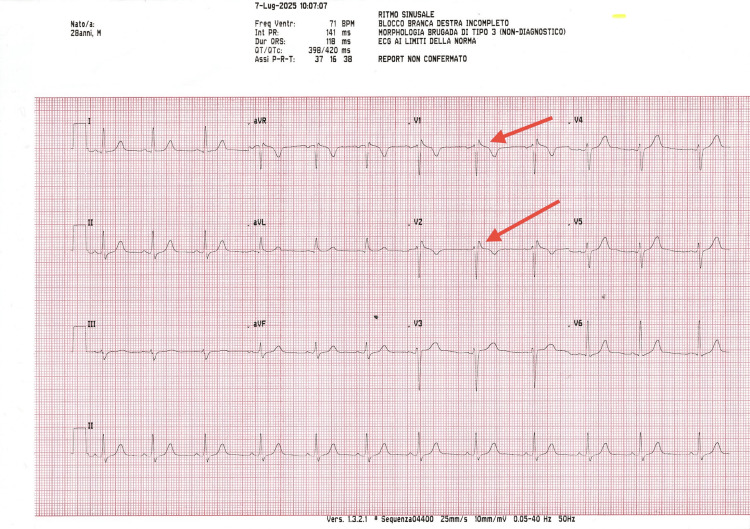
Preoperative 12-lead ECG recorded one day before the procedure showing ST-segment elevation in leads V1-V2 (red arrows). The tracing confirms normal PR interval, supporting clinical stability and procedural eligibility.

In addition, a further cardiologic consultation was arranged the day before the dental procedure, involving the cardiologist affiliated with our dental clinic, to independently assess the patient’s current clinical status and confirm procedural eligibility (Figure 2).

**Figure 3:**
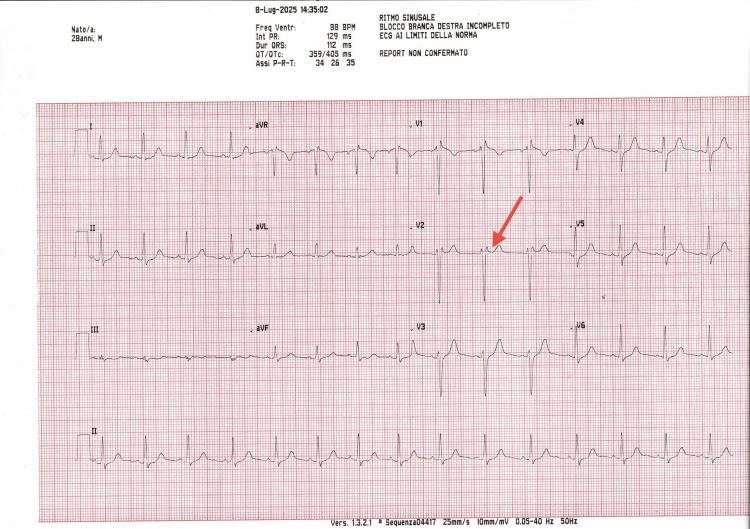
Baseline intraoperative 12-lead ECG obtained immediately before anesthetic administration. The tracing shows sinus rhythm with no acute changes from previous findings. The Brugada pattern shows a slightly saddleback shape in lead V2 (red arrow), consistent with a Brugada type 3 pattern.

**Figure 4:**
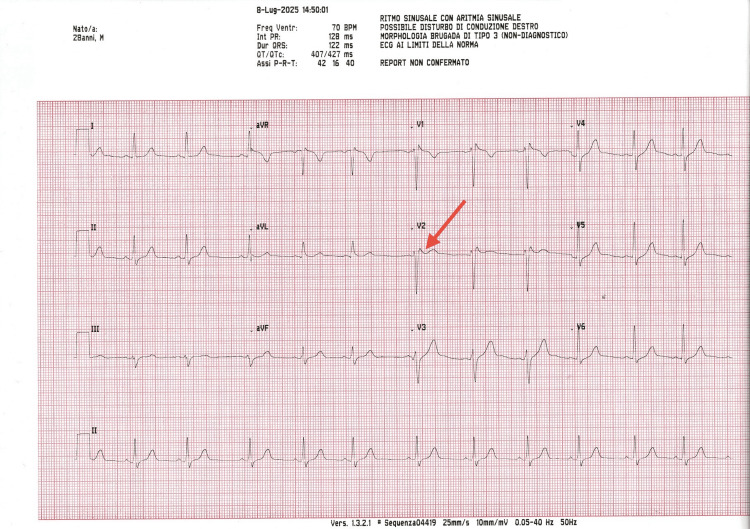
A 12-lead ECG recorded 15 minutes after infiltration of 4% articaine with epinephrine 1:100,000. No new ST-segment abnormalities are observed. This ECG also demonstrates the same Brugada pattern consistent with type 3, characterized by a slightly saddleback morphology (red arrow) in lead V2.

**Figure 5:**
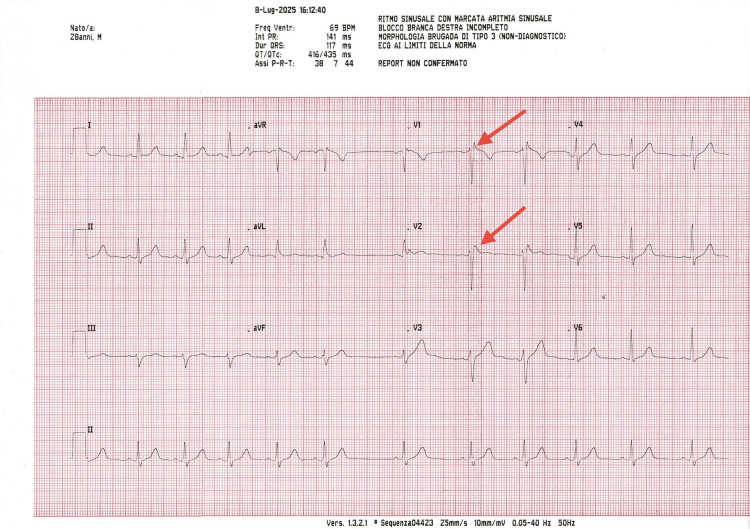
The final intraoperative ECG, recorded at the conclusion of the dental procedure, confirms persistent clinical stability throughout the session, with no significant modifications observed. The tracing consistently exhibits ST-segment elevation in leads V1-V2 (red arrows).

Despite the presence of the S-ICD, additional perioperative safety measures were deemed prudent given the patient’s high arrhythmic risk profile. These included continuous ECG and vital sign monitoring, conducted under the direct supervision of the cardiologist, who remained physically present and actively involved throughout the entire duration of the procedure. An automated external defibrillator (AED) capable of delivering biphasic shocks was positioned chairside as a precautionary measure in case of S-ICD malfunction or delayed activation during a malignant arrhythmic event. A written informed consent was provided to the patient several days prior to the procedure, allowing adequate time for review and consultation with his cardiologist or other specialists. On the day of the dental intervention, to assess the patient’s psychological readiness and determine whether anxiety could influence the procedure, a preoperative visual analog scale for anxiety was administered. The patient reported a moderate score of 4/10, indicating adequate emotional stability. Given the presence of an S-ICD and the identification of a periapical radiolucency on the preliminary intraoral radiograph of the tooth scheduled for endodontic treatment, antibiotic prophylaxis was prescribed. The patient received 2.0 g of oral amoxicillin 1 hour before the procedure, followed by 1 g every 12 hours for four additional days. After these preliminary steps, local anesthesia was administered using 4% articaine with epinephrine 1:100,000 (Articaina Ogna®, Giovanni Ogna & Figli Srl, Milan, Italy) via a standard infiltration technique at both the buccal and palatal sites. Half a cartridge (approximately 0.9 mL), equivalent to 36 mg articaine and 0.009 mg epinephrine, was injected incrementally and at a slow rate to minimize systemic absorption. In line with structured protocols proposed in the literature for patients with inherited cardiac channelopathies, a simplified yet cautious monitoring strategy was adopted. Given the pharmacokinetics of articaine, characterized by a rapid onset and an elimination half-life of approximately 20-40 minutes, with peak plasma concentrations typically reached within the first 15 to 30 minutes following infiltration, 12-lead ECG recordings were obtained at three critical time points: baseline (Figure 3), 15 minutes after local anesthetic administration to detect any early ECG changes during peak systemic exposure (Figure 4), and at the end of the dental procedure (Figure 5).

Blood pressure (BP) and oxygen saturation (SpO₂) were also measured at each phase to detect any transient hemodynamic changes: BP 120/70 mmHg and SpO₂ 98% at baseline, BP 120/70 mmHg and SpO₂ 97% 15 minutes after local anesthesia administration, and BP 130/80 mmHg and SpO₂ 98% at the end of the dental treatment. Following root canal treatment, the tooth was rebuilt using fiber posts and conservatively prepared for digital scanning. The patient tolerated the procedure uneventfully, with no ECG changes or hemodynamic instability. Continuous ECG monitoring was maintained throughout the session and extended for approximately 45 minutes postoperatively, in accordance with the cardiologist’s recommendations. The patient was discharged in stable condition after this observation period. A second clinical appointment, scheduled for the final cementation of the dental crown, did not require local anesthesia and was performed safely without cardiologic supervision, considering the non-invasive nature of the procedure and the absence of identified risk factors for arrhythmic events. Subsequent routine follow-up dental evaluations revealed no reported symptoms or complications.

## Discussion

BrS is a rare but clinically relevant cardiac condition that predisposes patients to ventricular arrhythmias and sudden cardiac death. It is recognized as one of the leading causes of sudden cardiac death in individuals under the age of 40. In affected patients, episodes of life-threatening arrhythmias may be triggered by various factors, including fever, vagal stimulation, certain medications, or procedural stress. For this reason, the perioperative management of BrS, particularly during anesthesia and surgery, requires specific precautions, including drug restrictions and continuous monitoring, to reduce the risk of arrhythmic events [8]. While local dental anesthesia is routinely administered in clinical practice, its use in BrS patients remains a matter of caution due to the potential proarrhythmic effects of sodium channel-blocking agents, particularly under stress or in the presence of additional triggers such as procedural pain, anxiety, or systemic drug absorption. According to BrugadaDrugs.org, the use of lidocaine or articaine for local dental anesthesia appears to be safe when combined with epinephrine (e.g., Xylocaine® with epinephrine or articaine/epinephrine formulations such as Ultracain® or Septanest® 1:100,000), provided that the administered dose is low and the anesthetic effect remains localized. As reported by Theodotou and Cillo, no significant adverse cardiac events have been observed in such settings, especially when systemic absorption is minimized through careful injection technique and proper patient monitoring [9]. A randomized, double-blind crossover pilot trial by Oliveira et al. investigated the safety of 2% lidocaine administered with and without epinephrine (1:100,000) in 12 BrS patients undergoing two separate sessions of restorative dental treatment; the use of local dental anesthesia with lidocaine, regardless of the use of a vasoconstrictor, did not result in life-threatening arrhythmias and appears to be safe in stable patients with cardiac channelopathies [10]. Building on this, a follow-up single-center study by the same group in 2023 further assessed the safety of local dental anesthesia in 12 implantable cardioverter-defibrillator recipients with various cardiac channelopathies, including BrS, long QT syndrome (LQTS), and catecholaminergic polymorphic ventricular tachycardia. Each patient underwent two monitored procedures using lidocaine 2% with and without epinephrine in a randomized crossover design. No life-threatening arrhythmias occurred, and neither heart rate, QTc interval, nor anxiety scores showed significant differences between the two anesthetic conditions. These findings reinforce the notion that, under controlled hospital conditions and with careful patient selection, lidocaine with or without epinephrine can be safely administered in this population. Both studies were performed exclusively in a hospital setting (Instituto do Coração - InCor, Hospital das Clínicas, University of São Paulo) under tightly controlled clinical conditions including extended Holter monitoring, ECG, blood pressure assessment, and continuous cardiology supervision [11]. Therefore, while the results are reassuring, they are not directly generalizable to private outpatient care, where logistical resources and specialist support may differ significantly. Although the studies by Oliveira et al. reported no adverse events associated with the use or omission of epinephrine, the addition of a vasoconstrictor to local anesthetic solutions is widely supported in the literature for its multiple clinical benefits. The vast majority of available studies consistently demonstrate that epinephrine contributes to prolonged anesthetic effect, enhances depth of anesthesia, and reduces intraoperative bleeding. Moreover, by reducing the rate of systemic absorption and peak plasma levels of the anesthetic agent, the vasoconstrictive component helps localize the anesthetic effect and allows for the use of lower total doses, thereby further minimizing potential cardiac risks, particularly in patients with arrhythmogenic conditions such as BrS [12]. In the present case, 4% articaine with 1:100,000 epinephrine was selected over lidocaine, based on evidence suggesting superior anesthetic efficacy, longer duration of action [13-14], and better intra- and postoperative pain control [15-16]. This decision was made in agreement with the attending cardiologist, taking into account the available literature and the recommendations from BrugadaDrugs.org, which currently lists only lidocaine and articaine among the local anesthetics considered acceptable in BrS patients. Although specific clinical data on articaine use in BrS patients remain limited, its selection was supported by published evidence indicating a favorable safety profile in electrophysiologically similar conditions, such as LQTS [17]. Therefore, this choice represented a carefully considered component of the overall anesthetic strategy adopted in this case. To the best of our knowledge, and in line with expert opinion, no adverse cardiac events have been reported in patients with BrS in connection with the use of local dental anesthesia, specifically articaine with epinephrine, provided that intravascular injection is avoided. This was further confirmed in private correspondence with Dr. Pieter G. Postema, MD, PhD, Cardiologist and Director of BrugadaDrugs.org (Amsterdam, The Netherlands), who emphasized the absence of known complications from articaine use in this population under standard conditions (email correspondence with the authors, June 2025). A similar view was independently expressed by Prof. Bortolo Martini, MD, Cardiologist (Vicenza, Italy), who noted that no adverse cardiac events have ever been reported worldwide in connection with dental treatment in BrS patients, cautioning against generating undue alarm in routine clinical settings (email correspondence with the authors, July 2025). This aligns with prior published positions expressed by the same experts. While expert opinion represents the lowest level of evidence within the hierarchy of evidence-based medicine, it remains a recognized component of clinical reasoning, particularly in areas where high-quality studies are lacking.

Ethical and regulatory considerations

In accordance with the ASA Physical Status Classification System (last updated 2020), patients with severe systemic disease and an implanted pacemaker are classified as ASA III. Although pacemakers and S-ICDs serve different roles, both reflect a significant underlying cardiac pathology. In patients with BrS, the presence of an S-ICD therefore supports classification as ASA III, even in the absence of active symptoms. The ASA itself states that this classification is designed to assess and communicate a patient’s pre-anesthesia medical comorbidities. While the ASA score does not independently predict perioperative risk, it can help estimate that risk when interpreted alongside other factors such as the type of procedure, patient frailty, and clinical stability. In this context, classifying BrS patients with an S-ICD as ASA III is not a discretionary or subjective decision but a justified application of current standards. However, this classification does not automatically contraindicate outpatient dental procedures, particularly when sedation is not employed. In such cases, ASA III should be interpreted within the procedural context, guided by clinical judgment, specialist input, and appropriate safeguards. This has important implications when assessing the appropriateness of outpatient settings for dental procedures in such patients and underscores the need for a multidisciplinary, patient-specific approach to risk stratification and procedural planning. This ethical imperative is consistent with Article 14 of the Italian Code of Medical Ethics, titled “Prevention and Management of Adverse Events and Safety of Care”, which states: “The physician shall act with the aim of ensuring the highest possible standards of safety for both the patient and the healthcare team, promoting the adaptation of organizational processes and professional conduct accordingly, and contributing to the prevention and management of clinical risk.” This reinforces the importance of individualized planning and interprofessional collaboration, especially when managing medically complex patients in outpatient dental settings. This consideration becomes even more relevant in light of recent findings showing that, although ASA classification has traditionally been assigned by anesthesiologists, surgeons often apply ASA classes during preoperative planning. Kwa et al. confirmed that this practice is already widespread across surgical settings [18]. In the dental management of patients with BrS, a multidisciplinary approach involving both physicians and dental professionals is strongly recommended. In settings where only local anesthesia is used without sedation, such as in the present case, the ASA classification should be interpreted with caution, based on the patient’s current functional status, clinical stability, and the invasiveness of the dental procedure. With all due caution, and acknowledging the inherent limitations of such an approach, it may therefore be reasonable to consider that, in the absence of anesthesiologist involvement, the collaborative attribution of an ASA class by the cardiologist and the treating dentist may serve as a pragmatic tool for preoperative risk assessment in dental settings. In the present case, legal and ethical consultation with the local Italian Medical Council confirmed that, when appropriate precautions are in place, such as individualized cardiologic clearance, intra-procedural ECG monitoring, and the use of authorized outpatient dental facilities, the intervention may be conducted safely outside the hospital setting. In light of medico-legal concerns regarding the suitability of outpatient dental treatment in patients with BrS, the authors of this manuscript formally submitted a request for clarification to the Italian Board of Dentists (Commissione Albo Odontoiatri [CAO]) on June 15, 2025. In their official reply, the CAO confirmed the absence of any national regulation prohibiting such treatment and stated that all dental clinics are authorized to treat patients regardless of pre-existing medical conditions, provided that clinical judgment ensures appropriate safety standards (CAO Official Reply received June 25, 2025; available upon request). This position is further supported by the real-world evidence that the vast majority of BrS patients, including S-ICD carriers, routinely undergo dental care in non-hospital environments. Such an approach respects both patient autonomy and clinical prudence, avoiding undue barriers to necessary dental treatment while maintaining appropriate safeguards in line with current regulatory expectations and risk management standards. Nonetheless, from a legal standpoint, it is important to emphasize that a multidisciplinary and prudently structured approach, while ethically sound and clinically appropriate, does not entirely eliminate the possibility of medico-legal liability for the dentist, particularly in outpatient settings. Even when the patient has been thoroughly evaluated and cleared by a cardiologist, the responsibility for the procedure may ultimately fall on the practitioner who performs it. In this context, it is essential to clarify that the legal management of a medical claim, whether through extrajudicial negotiation or formal judicial proceedings, is not determined by the setting of care (hospital vs. outpatient) but rather by the subsequent evolution of the dispute itself. In all cases, the patient must first demonstrate the occurrence of harm and its causal connection to the healthcare intervention. The burden then shifts to the provider, who must prove adherence to recognized standards of care, applicable clinical guidelines, or the occurrence of an unforeseeable event that justifies exemption from liability. While the distinction between judicial and extrajudicial paths is not inherently tied to the treatment environment, it is worth noting that, in practical terms, claims arising from private outpatient care are more often managed through extrajudicial channels, such as direct negotiation or mediation. This trend likely reflects the more personalized nature of care, the absence of institutional legal frameworks, and the generally lower complexity of the procedures involved. Therefore, even when conducted with the utmost care and interdisciplinary coordination, outpatient procedures in high-risk patients, such as those with BrS, continue to carry a residual medico-legal risk. In such contexts, thorough documentation, compliance with protocols, and the acquisition of clear and informed consent represent not just ethical imperatives but also critical legal protections for the treating dentist. The present case reflects an effort to mitigate these risks through detailed documentation, informed consent, real-time monitoring, and interdisciplinary collaboration. In such contexts, treatment in a private outpatient dental clinic may be ethically and clinically appropriate, as long as adequate cardiologic clearance, procedural planning, and perioperative precautions are implemented. According to Domingues et al., in high-risk patients such as those with BrS, continuous ECG monitoring during both the intraoperative and immediate postoperative phases is not simply recommended but essential to ensure timely detection and management of potential arrhythmic events [19].

Ethical foundations of informed consent

To promote transparency and shared decision-making, the written informed consent form was provided to the patient several days prior to the scheduled procedure. This allowed sufficient time for personal reflection and consultation with trusted healthcare professionals, including the referring cardiologist. In line with current ethical and medico-legal recommendations for managing complex cardiovascular patients in outpatient settings, the consent process specifically emphasized the rationale for the chosen safety measures. In particular, the patient was clearly informed about the critical importance of continuous cardiologic monitoring during local anesthetic infiltration, including the cardiologist’s role in detecting and responding promptly to any ECG abnormalities.

Anxiety management

Finally, we underline the relevance of proactive anxiety management, both pharmacological and non-pharmacological, to reduce sympathetic activation and mitigate arrhythmogenic risk in BrS patients, particularly during stressful procedures. Anxiety may act as a potential trigger for arrhythmias by amplifying autonomic imbalance, especially in patients already sensitized by the psychological burden of their diagnosis. Recent literature highlights that up to one in six BrS patients may develop clinically significant anxiety or depression following diagnosis or S-ICD implantation, and a relevant proportion exhibit type D personality traits, characterized by chronic negative affectivity and social inhibition, which may further impair emotional regulation and stress resilience [20]. Early identification of psychological vulnerability and timely support may therefore play a complementary role in ensuring perioperative safety.

Suggested protocol for non-hospital dental management of BrS

Although high-level scientific studies and strong evidence-based guidelines are currently lacking, particularly in private dental practice settings, the following precautionary considerations are proposed, drawing on current literature and practical insights from the present case, to support risk-aware dental management of patients with BrS: (1) preoperative cardiologic evaluation, including thorough documentation of the patient’s arrhythmic risk profile, in collaboration with the patient’s referring cardiologist; (2) presence of a cardiologist experienced in the perioperative management of cardiac channelopathies, responsible for continuous ECG assessment and prepared to promptly identify potential arrhythmic abnormalities; (3) chairside availability of external defibrillator and emergency equipment; (4) selection of regional anesthetic agents with an established safety profile in BrS patients, such as lidocaine or articaine with epinephrine (1:100,000); (5) use of the lowest effective dose of local anesthetic, administered slowly and incrementally, with careful aspiration to minimize systemic absorption; (6) continuous intraoperative and postoperative monitoring; and (7) proactive anxiety management, using pharmacologic and/or non-pharmacologic strategies, to mitigate stress-induced arrhythmic risk.

## Conclusions

This case suggests that dental procedures involving local anesthesia may be safely performed in patients with BrS, even within a private dental clinic, provided that individualized, interdisciplinary planning and real-time cardiologic monitoring are implemented. The favorable outcome following the administration of 4% articaine with 1:100,000 epinephrine supports its cautious use in carefully selected patients. Although this report is limited to a single observation, it outlines a structured protocol that may be replicated in similar clinical contexts, potentially contributing to safer outpatient dental care for individuals with cardiac channelopathies. These preliminary findings highlight the importance of safety, access to care, and individualized clinical judgment in managing BrS patients undergoing dental procedures. Further studies involving larger cohorts are needed to confirm the reproducibility of this approach and to develop evidence-based protocols applicable in outpatient settings.
